# CAFS: An Attention-Based Co-Segmentation Semi-Supervised Method for Nasopharyngeal Carcinoma Segmentation

**DOI:** 10.3390/s22135053

**Published:** 2022-07-05

**Authors:** Yitong Chen, Guanghui Han, Tianyu Lin, Xiujian Liu

**Affiliations:** 1 School of Biomedical Engineering, Sun Yat-sen University, Shenzhen 518107, China; chenyt263@mail2.sysu.edu.cn (Y.C.); hangh3@mail.sysu.edu.cn (G.H.); linty6@mail2.sysu.edu.cn (T.L.); 2 School of Information Engineering, North China University of Water Resources and Electric Power, Zhengzhou 450046, China

**Keywords:** nasopharyngeal carcinoma, deep learning, semi-supervision

## Abstract

Accurate segmentation of nasopharyngeal carcinoma is essential to its treatment effect. However, there are several challenges in existing deep learning-based segmentation methods. First, the acquisition of labeled data are challenging. Second, the nasopharyngeal carcinoma is similar to the surrounding tissues. Third, the shape of nasopharyngeal carcinoma is complex. These challenges make the segmentation of nasopharyngeal carcinoma difficult. This paper proposes a novel semi-supervised method named CAFS for automatic segmentation of nasopharyngeal carcinoma. CAFS addresses the above challenges through three mechanisms: the teacher–student cooperative segmentation mechanism, the attention mechanism, and the feedback mechanism. CAFS can use only a small amount of labeled nasopharyngeal carcinoma data to segment the cancer region accurately. The average DSC value of CAFS is 0.8723 on the nasopharyngeal carcinoma segmentation task. Moreover, CAFS has outperformed the state-of-the-art nasopharyngeal carcinoma segmentation methods in the comparison experiment. Among the compared state-of-the-art methods, CAFS achieved the highest values of DSC, Jaccard, and precision. In particular, the DSC value of CAFS is 7.42% higher than the highest DSC value in the state-of-the-art methods.

## 1. Introduction

Nasopharyngeal carcinoma [1,2] is one of the most common cancers, wildly occurring around the world. According to global cancer statistics, there were 133,354 new nasopharyngeal carcinoma cases and 80,008 deaths in 2020 [3]. Nasopharyngeal carcinoma is an epithelial carcinoma arising from the nasopharyngeal mucosal lining [4], which is generally observed at the pharyngeal recess of the nasopharynx [5]. In the clinic, nasopharyngeal carcinoma has three types: ascending, descending, and mixed [6]. The ascending type invades the skull base crania and destroys nerves, the descending type metastasizes to distant tissues through cervical lymph, and the mixed type has both. Thus, due to the particular location of nasopharyngeal carcinoma, it is abnormally dangerous once it metastasizes.

Currently, radiotherapy has become one of the most effective methods for treating nasopharyngeal carcinoma [7]. The segmentation of nasopharyngeal carcinoma images significantly affects the effects of radiotherapy [8]. Accurate segmentation would improve the effectiveness of radiotherapy and thus increase patient survival [9]. The traditional method of segmentation is manually operated by the physician. However, due to the irregularity of nasopharyngeal carcinoma tissues, it is often a time-consuming burden for doctors to manually segment the boundaries [10]. Moreover, manual segmentation is often so subjective that doctors with different levels of expertise may come up with different segmentation results [11].

To reduce the burden on physicians, more and more deep learning algorithms are now being utilized to segment medical images [12,13,14]. However, it is difficult for many deep learning models to segment nasopharyngeal carcinoma boundaries accurately. First, lots of deep learning algorithms typically utilize the fully-supervised approach. The fully-supervised approach is that all training data are labeled and the model is trained using these labeled data [15]. This means that the model requires a large amount of labeled data to obtain the expected training results [16]. However, the hardship of annotating interested targets hinders fully-supervised learning in medical imaging. In contrast, unlabeled data are readily available [17]. Second, the imaging characteristics of nasopharyngeal carcinoma usually resemble the surrounding tissue [18,19], making it challenging to identify. That leads many algorithms to mistake the surrounding tissue for nasopharyngeal carcinoma. Third, due to the irregular shape of the nasal cavity, the shape of nasopharyngeal carcinoma is usually very complex as well [20,21], which leads to many algorithms that do not segment the boundaries accurately.

**Figure 1:**
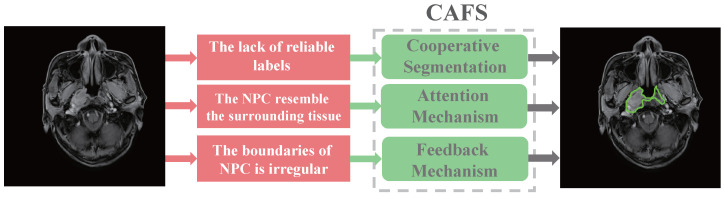
The task of CAFS is to automatically segment out the nasopharyngeal carcinoma boundaries by using only a small amount of labeled data. However, there are several challenges of segmenting nasopharyngeal carcinoma. First, reliable labeled data are difficult to obtain. Second, the nasopharyngeal carcinoma resembles the surrounding tissue. Third, the boundaries of nasopharyngeal carcinoma are irregular. The CAFS utilizes the cooperative, attention mechanism, and the feedback mechanism to address these difficulties, respectively.

To address the challenges encountered in the above-mentioned conventional methods of fully-supervised segmentation of nasopharyngeal carcinoma, and therefore to improve the efficacy and survival rate of nasopharyngeal carcinoma, this paper proposes an attention-based co-segmentation semi-supervised method named CAFS for automatic segmentation of nasopharyngeal carcinoma. The semi-supervised approach means that only a portion of the training data contains labels, and uses these labeled and unlabeled data to train the model collaboratively [22]. As shown in Figure 1, CAFS contains three primary strategies: the teacher–student cooperative segmentation mechanism, the attention mechanism, and the feedback mechanism. The teacher–student model is typically used in knowledge distillation [23]. In general, the teacher model uses the obtained knowledge to guide the student model training, making the student model have comparable performance to the teacher model. Among CAFS, the teacher–student cooperative segmentation mechanism aims to reduce the number of nasopharyngeal carcinoma labels used. The teacher model learns from a small amount of labeled nasopharyngeal carcinoma data and then generates pseudo-masks for the unlabeled nasopharyngeal carcinoma data. The student model utilizes the unlabeled nasopharyngeal carcinoma data and the pseudo-mask generated by the teacher model to train itself and segment the unlabeled nasopharyngeal carcinoma data. This allows for reducing the use of labeled data. The attention mechanism serves to pinpoint the location of cancer, which zooms in on the target and thus captures more information to localize the nasopharyngeal carcinoma. The feedback mechanism aims to make the segmentation boundaries of nasopharyngeal carcinoma more accurate. The student model is trained on unlabeled data and pseudo-masks and then predicts the labeled data. The prediction results are compared with the ground truth to generate feedback to update the model’s parameters. We trained and validated the performance of CAFS on 3555 nasopharyngeal carcinoma images. The results demonstrate that CAFS performs well in segmenting the nasopharyngeal carcinoma boundaries. CAFS achieved a DSC value of 0.8723, a Jaccard value of 0.7964, a precision value of 0.8849, and a recall value of 0.8796. In addition, we also compare CAFS with state-of-the-art nasopharyngeal carcinoma segmentation methods. In the comparison experiments, CAFS achieved the leading segmentation level, which obtained the highest values of DSC, Jaccard, and precision. In addition, the CAFS segmentation out of the nasopharyngeal carcinoma boundary compared with four other segmentation models is closest to the ground truth.

In general, the main contribution of CAFS are as follows:

- The teacher–student cooperative segmentation mechanism allows CAFS to segment nasopharyngeal carcinoma using only a small amount of labeled data;
- The attention mechanism could prevent confusing nasopharyngeal carcinoma with surrounding tissues;
- The feedback mechanism allows CAFS to segment nasopharyngeal carcinoma more accurately.

## 2. Related Work

In this section, related studies on Nasopharyngeal Carcinoma segmentation are categorized into fully-supervised methods and semi-supervised methods, which are to be introduced, respectively.

### 2.1. Fully-Supervised

The most common method for the automatic segmentation of nasopharyngeal carcinoma is the fully-supervised methods [24,25,26,27,28]. In the last few decades, deep learning methods have been increasingly used in medical image segmentation [29,30,31]. Among them, many fully supervised algorithms have been proposed for nasopharyngeal carcinoma segmentation. Convolutional neural networks (CNN) [32] are an effective image segmentation method that captures contextual semantics by computing high-level feature maps [33,34]. Since the pioneering CNN algorithm by Lecun et al., in 1990, more and more improved CNN algorithms for image segmentation have been proposed. Pan et al. [35] improved the typical CNN network by designing dilated convolution at each layer of the FPN to obtain contextual associations, which was applied to nasopharyngeal organ target segmentation. Some other scholars segment nasopharyngeal carcinoma by improving CNN into the CNN-based method with three-dimensional filters [36,37,38]. Ronneberger et al. [39] propose in 2015 a convolutional networks called U-Net for biomedical image segmentation. After that, many segmentation algorithms for medical images were adapted from U-Net. Some scholars combined mechanisms such as attention mechanism and residual connectivity with U-Net to improve segmentation performance and segment the nasopharyngeal carcinoma [40,41,42]. In order to accommodate the volume segmentation of medical images, many U-Net-based 3D models have been developed as well [43,44]. While these fully supervised methods are capable of achieving the excellent segmentation effect, they predicate by using a large amount of labeled data. The fact that reliable labeled data are often tough to obtain as specialized medical knowledge and time are both demanded.

### 2.2. Semi-Supervised

More and more semi-supervised segmentation methods have been proposed in recent years to confront the challenge of difficult access to annotated data [45]. Self-training is one of the most commonly used semi-supervised methods [46]. It first trains using a small amount of labeled data, then makes predictions on unlabeled data, and finally mixes the excellent predictions with labeled data for training [47,48]. Another common semi-supervised method is co-training, which uses the interworking between two networks to improve the segmentation performance [49,50]. Hu et al. [51] proposed uncertainty and attention guided consistency semi-supervised method to segment nasopharyngeal carcinoma. Lou et al. [52] proposed a semi-supervised method that extends the backbone segmentation network to produce pyramidal predictions at different scales. Zhang et al. [53] then use the teacher’s uncertainty estimates to guide the student and perform consistent learning to uncover more information from the unlabeled data.

Sun et al. [54] also applies the teacher–student paradigm in medical image segmentation. It is worth noting that the mixed supervision in [54] stands for partial dense-labeled supervision from labeled datasets and supplementary loose bounding-box supervision for both labeled and unlabeled data. Our work only uses partial dense-labeled supervision. In addition, Ref. [54] applies bounding-box supervision to provide localization information. In our work, the attention mechanism involves localizing the nasopharyngeal carcinoma target. Moreover, Sun et al. [54] have the teacher model well-trained before providing pseudo label guidance for the student, while we optimize both teacher and student models simultaneously.

In addition to the self-training and the co-training, semi-supervised methods include paradigms such as generative models, transductive support vector machines, and Graph-Based methods as well.

## 3. Methodology

**Figure 2:**
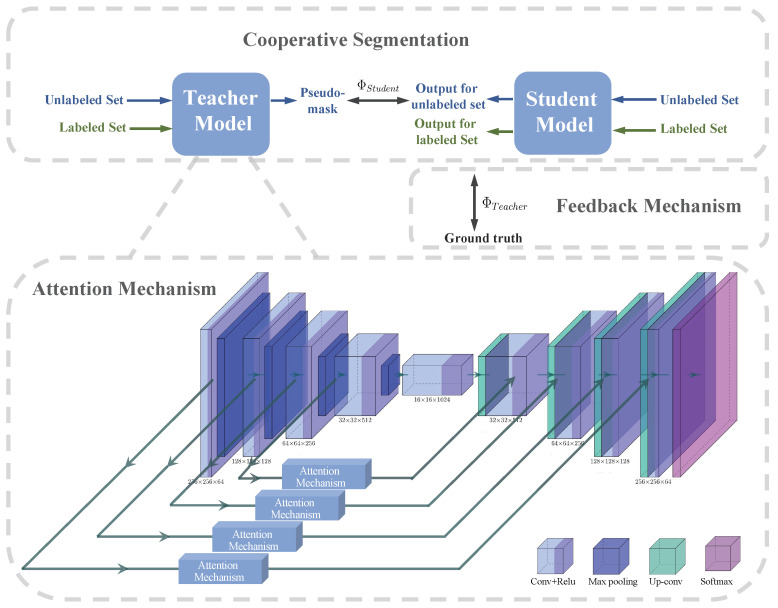
This figure shows the composition of CAFS. As shown in the figure, CAFS consists of three main parts, which are the teacher–student cooperative segmentation mechanism, the attention mechanism, and the feedback mechanism. The teacher–student cooperative segmentation mechanism includes both teacher and student models, each of which uses U-Net2D as the backbone network. The attention mechanism is fused between the skip connection of the backbone U-Net. CAFS is a novel semi-supervised segmentation method, which incorporates several impelling strategies to address the difficulty of segmenting nasopharyngeal carcinoma and make the segmentation more effective.

CAFS contains three primary strategies: the teacher–student cooperative segmentation mechanism, the attention mechanism, and the feedback mechanism, as shown in Figure 2. To address the challenges in the segmentation of nasopharyngeal carcinoma, each mechanism plays an important role respectively.

### 3.1. Cooperative Segmentation

CAFS contains two models, the teacher and the student models, which cooperate to achieve the segmentation task using a small amount of nasopharyngeal carcinoma labeled data. Although both these two models use U-Net as the structure, the parameters of each are independent of each other at the beginning of training. First, the teacher model is trained by a small amount of labeled data so as to update the parameters $\Phi_{Teacher}$ of the teacher model. The objective of the training is the Cross-Entropy Loss Function (CELF). The $Y_{GT}$ refers to the ground truth, and the ${\hat{Y}}_{Teacher}$ refers to the prediction of the teacher model:

(1) $$\Phi_{Teacher}∼L_{Cross-Entropy}=-\sum_{k=1}^{N}\left(Y_{GT}\times \ln{\hat{Y}}_{Teacher}\right)$$

When the training has reached the expectation, the unlabeled nasopharyngeal carcinoma data $X_{unlabeled}$ are fed into the teacher model $\Phi_{Teacher}$ to obtain the predicted segmentation pseudo-mask ${\hat{Y}}_{pseudo-mask}$ for unlabeled nasopharyngeal carcinoma data.

The same batch of unlabeled data $X_{unlabeled}$ is then fed into the student model and utilizes the generated pseudo-mask ${\hat{Y}}_{pseudo-mask}$ as the supervision, thus updating the parameters $\Phi_{Student}$ of the student model. The CELF is used as the objective for training the student model as well. The ${\hat{Y}}_{Student}$ refers to the prediction of the student model:

(2) $$\Phi_{Student}∼L_{Cross-Entropy}=-\sum_{k=1}^{N}\left({\hat{Y}}_{pseudo-mask}\times \ln{\hat{Y}}_{Student}\right)$$

The final prediction ${\hat{Y}}_{output}$ of nasopharyngeal carcinoma segmentation was generated by the student model:

(3) $$X_{input}\;\overset{\;\;\Phi_{Student}\;\;}{\to }\;{\hat{Y}}_{output}$$

The teacher and student models cooperate to make CAFS require only a small amount of labeled nasopharyngeal carcinoma data to complete the segmentation task.

### 3.2. Attention Mechanism

The function of the attention mechanism [55] is to sift through the many pieces of information that are effective for segmenting nasopharyngeal carcinoma.

In recent years, more and more attention mechanisms have been used in the field of computer vision, such as the external attention mechanism proposed by Guo et al. [56], the self-attention mechanism proposed by Vaswani et al. [57], and the Bottleneck Attention Module proposed by Park et al. [58]. These attention mechanisms play a role in image segmentation to extract information about the details of the target, but they are not dynamically adjustable when used. However, the fact that, in the neuroscience community, the receptive field size of the visual cortex neurons is regulated by the stimulus [59]. Thus, CAFS introduces the adjustable attention mechanism [60]. Thus, it can flexibly deal with the change of target due to the difference in scale during the downsampling.

CAFS fuses the adjustable attention mechanism to the skip connection of the U-Net. The input of the attention mechanism is the feature map output from the encoder downsampling layer, and the output is in the upsampling decoder layer.

Firstly, the input feature map *X* is transformed by grouped/depthwise convolutions, batch normalization, and ReLU function by using several convolution kernels of particular sizes. Then, different intermediate transformations $\bar{U}$, $\tilde{U}$, $\hat{U}$ are obtained for different sizes of convolution kernels, respectively.

The results of the transformation of these different sizes of branches are then added together as follows:

(4) $$U=\bar{U}+\tilde{U}+\hat{U}$$

Then, use global average pooling to process the resulting *U*, which generates the channel-wise statistics $S_{channel}$. The channel-wise statistics are then processed through the batch normalization and ReLU functions in turn to obtain a compressed feature map *m*:

(5) $$m=F_{ReLU}\left(\left(F_{BN}\left(S_{channel}\right)\right)\right)$$

Then, calculate the weights of each of the these branches respectively:

(6) $$\alpha =\frac{e^{a\times m}}{e^{a\times m}+e^{b\times m}+e^{c\times m}},\;\left(a,\;b,\;c\in R\right)$$

Finally, the attention map is obtained based on the intermediate transformation results of the above these branches and their weights:

(7) $$Y=\alpha \bar{U}+\beta \tilde{U}+\gamma \hat{U}$$

Since feature maps of different scales generate different weights for different branches in Equation (6), this is equivalent to a gate that controls the final attention vector generation depending on the scale. Therefore, the attention mechanism adaptively generates attention vectors for output to the upsampling layer under different scale feature map stimuli generated by different downsampling layers.

CAFS fuses the attention mechanism, which enlarges the target area and reduces the background area in the nasopharyngeal carcinoma image while keeping the image size constant. Ultimately, more information can be captured by enlarging the target area and avoiding the confusion of the nasopharyngeal carcinoma area with the surrounding tissues during segmentation.

### 3.3. Feedback Mechanism

CAFS introduces a feedback mechanism [61], which allows for more accurate segmentation of nasopharyngeal carcinoma boundaries. As stated in Section 3.1, the student model was trained on unlabeled nasopharyngeal carcinoma data and the pseudo-masks to update the parameters. Now have the student model make predictions on labeled nasopharyngeal carcinoma data $X_{labeled}$, and the predictions ${\hat{Y}}_{pred}$ then compare with the ground truth $Y_{GT}$ utilizing the CELF:

(8) $$L_{Cross-Entropy}=-\sum_{k=1}^{N}\left(Y_{GT}\times \ln{\hat{Y}}_{pred}\right)$$

The comparison results are fed back to the teacher model and then updates the parameters of the teacher model. The ${\Phi^{\prime }}_{Teacher}$ refers to the updated parameters of the teacher model.

The prediction results of the student model for the labelled nasopharyngeal cancer data were determined by the parameters of the student model, which relied on the pseudo-label generated by the teacher model for optimisation. The feedback obtained from comparing the prediction results of the student model with the ground truth guides the teacher model to generate improved pseudo-masks, which in turn leads to the generation of more effective student model parameters and more accurate predictions of the nasopharyngeal carcinoma boundaries.

## 4. Experiments

### 4.1. Data

The data for this paper were obtained from 83 patients who were diagnosed with nasopharyngeal carcinoma. All patients were scanned by Siemens Aera MRI system on their heads and neck, and contrast was used to enhance the display during the scanning process. We stored the scanned MRI images in Digital Imaging and Communications in Medicine (DICOM) file format. We used the annotation tool itk-snap for 831 images containing nasopharyngeal carcinoma from these 83 patients to annotate and store them as 2D image files. We applied the data augmentation method to augment the datasets. Data augmentation methods include rotating, flipping, and mirroring the images. After augmentation, the number of the images is augmented from the original 831 images to 3555 images. Our data are divided according to patients, though the number of slices included per patient is not exactly equal. We used 57 patients as the training set, 13 patients as the training set, and 13 patients as the test set in a ratio of about 70%:15%:15%. This means the data of the same patient can only appear in the training set or the test set, which avoid several consecutive slices in the same patient appearing in the training and test sets respectively and affect the results. In addition, we cropped the area outside the nasal cavity to reduce the computational burden and resized the image to 256×256.

### 4.2. Implementation Details

As described in Section 3.1, we use the U-Net as the network framework, and the parameters of the teacher and the student are independent of each other at the beginning of training. The implementation of CAFS is based on the PyTorch library, which we run on one RTX 3090 GPU. Before formally starting the training, the teacher model should first be pretrained to excellence. We conducted sufficient experiments on the validation set to ultimately determine the optimal set of hyperparameter values by altering the values of the hyperparameters (Table 1) and observing the segmentation results on the validation set. CAFS chooses SGD as the optimizer for both pretraining and the following segmentation training, the weight decay of which is 0.0001. The learning rate is 0.001 for training. The training iterations are 1500. The batch size is 8. In our experiments, we selected 1000 of the total 2874 images in the training set as labeled data and the remaining 1974 as unlabeled data. The experimental results also show that such an allocation achieves the most optimal segmentation performance. Moreover, in the attention mechanism, we use four convolutional kernels of different sizes: 1 × 1, 3 × 3, 5 × 5, and 7 × 7.

### 4.3. Evaluation Metrics

In this paper, the following metrics are used for evaluation. These evaluation metrics are also used in the subsequent comparison experiments.

DiceSimilarityCoefficient(DSC) is used to measure the similarity of two sets, the value range is [0, 1], the larger the value, the more similar the two sets:

(9) $$DSC=\frac{2|A\cap B|}{\left|A|+|B\right|}$$

Jaccardcoefficient is used to calculate the similarity between two sets, defined as the ratio of the size of the intersection of *A* and *B* to the size of the union of *A* and *B*. The value range is [0, 1], the larger the value, the more similar the two sets:

(10) $$J\left(A,B\right)=\frac{\left|A\cap B\right|}{\left|A\cup B\right|}$$

Precision is specific to the prediction results, and it indicates how many of the samples with positive predictions are actually positive samples:

(11) $$precision=\frac{TP}{TP+FP}$$

where *TP* and *FP* are the numbers of true positive and false positive pixels across all images in the valid set and test set.

Recall is for the original sample, and it indicates how many positive cases in the sample were predicted correctly:

(12) $$recall=\frac{TP}{TP+FN}$$

where *TP* and *FN* are the numbers of true positive, and false negative pixels across all images in the valid set and test set.

## 5. Results

### 5.1. Performance of CAFS

**Figure 3:**
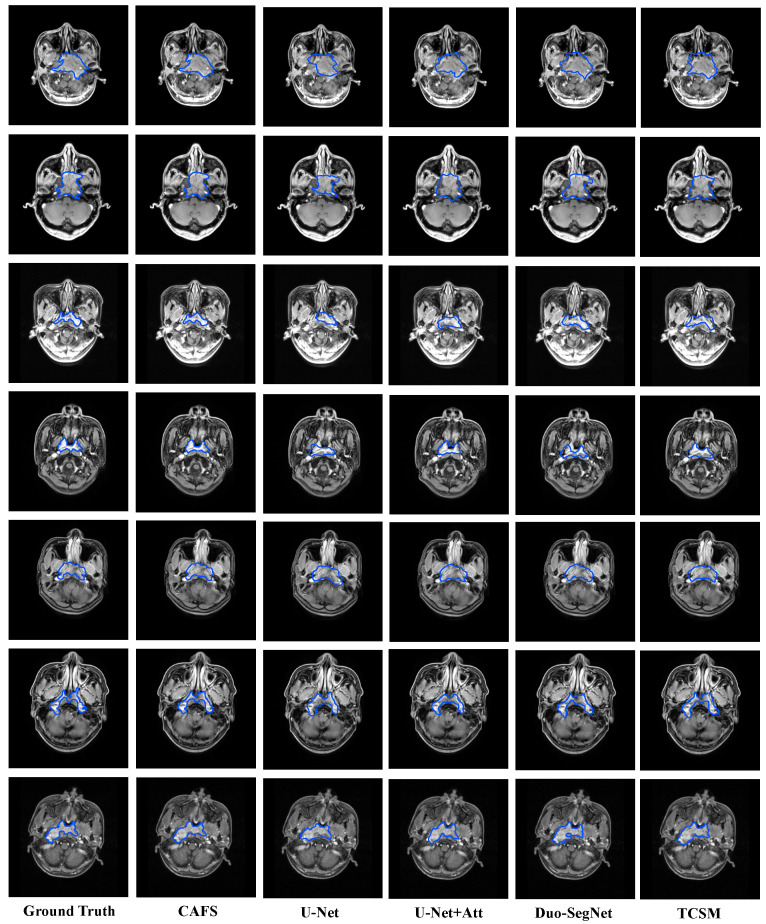
Segmentation results of five models. We selected several representative images of nasopharyngeal carcinoma from the test set. These images are characterized by the similarity of the nasopharyngeal carcinoma to the surrounding tissue (rows 1, 3, 7) and the complexity of the nasopharyngeal carcinoma boundaries (rows 2, 4, 5, 6). From left to right, they are ground truth, CAFS, U-Net, U-Net + Attention, Duo-SegNet, and TCSM. The blue line in the figure represents the boundaries of each model nasopharyngeal carcinoma segmentation result.

As we elaborated in Section 2, the teacher model plays a guiding role in the training process and the final prediction of CAFS is carried out by the student model. Therefore, the results in Section 5 are generated by the student model. The results show that CAFS performs excellently on the nasopharyngeal carcinoma segmentation task. The final test metrics: The average DSC value is 0.8723, the average Jaccard coefficient is 0.7964, the average precision is 0.8849, and the average recall is 0.8796. In addition, the average specificity is 0.9447. The displayed metrics and segmentation results illustrate that CAFS can overcome the nasopharyngeal carcinoma segmentation challenges presented in Section 1. First, the implementation of our method uses only a small amount of labeled nasopharyngeal carcinoma data for training. Second, Figure 3 (rows 1, 3, 7) show that, in most cases where nasopharyngeal carcinoma resembles surrounding tissues and is easily confused, CAFS can still accurately segment nasopharyngeal carcinoma from them. Third, Figure 3 (rows 2, 4, 5, 6) shows that CAFS can still do well in the case of irregular nasopharyngeal carcinoma boundaries.

We explored the effect of the amount of labeled data on the experimental results. In the experiment, we alter the amount of labeled data in the training set and observe the metric changes in the results. The training set has a total of 2874 images. We set the number of labeled data to 500, 1000, 1500, and 2000, respectively. The comparison results are shown in Table 2. The experimental results showed that the best results were obtained when the number of labeled data was 1000. It is worth being noticed that Table 2 shows that it is not the case that the more labeled data are used, the better the model segmentation is. The training set contains labeled data and unlabeled data. The teacher model generates pseudo-masks for the unlabeled data in the training set. The student model utilizes the unlabeled data and the pseudo-masks as supervision to update the parameters. If the proportion of labeled data in the training set increases, the proportion of unlabeled data decreases, and fewer data can be used to train the student model, thus the performance of the student model decreases. On the other hand, the decrease in the performance of the student model generates false feedback which misleads the teacher model to generate a worse pseudo-mask.

In addition, to further illustrate the superior feature extraction performance of the adaptive attention mechanism, we conducted comparison experiments with four other state-of-the-art attention mechanisms. In the comparison experiments of attention mechanisms, we fused each attention mechanism into the skip connection layer of the teacher network and the student network, respectively, with the rest of the model remaining unchanged. The four metrics in Section 4.3 are still used to evaluate the performance, and the performance of the model fusing different attention mechanism is shown in Table 3. It can be observed that the adaptive attention mechanism achieves optimal values for all metrics except recall. This indicates that the adaptive attention mechanism has a superior performance.

### 5.2. Ablation Analysis

Through extensive experiments, we performed ablation analysis to demonstrate the contribution of the modules proposed. CAFS consists of three modules: Cooperative segmentation (C-S), Attention mechanism (Att), and Feedback mechanism (Fb). In this section, we eliminate these three modules in turn and conduct experiments according to the following module combinations: the complete CAFS, removing the Attention mechanism leaving Cooperative segmentation and Feedback mechanism, removing the Feedback mechanism leaving Cooperative segmentation and Attention mechanism, only Cooperative segmentation, and the CAFS without pretraining. The experimental results are shown in Table 4.

The results demonstrate that CAFS performs the worst when only Cooperative segmentation is utilized. Therefore, the attention mechanism and feedback mechanism play an important role in the segmentation of nasopharyngeal carcinoma, which can effectively improve the model performance. For the metric DSC, removing the Feedback mechanism by leaving the Cooperative segmentation and Attention mechanism works better than removing the Attention mechanism by leaving the Cooperative segmentation and Feedback mechanism. Thus, for the DSC, the contribution of the attention mechanism may be greater than the contribution of the feedback mechanism. In addition, notice that, if the teacher model is not pre-trained, the segmentation results of the network will drop sharply. Therefore, pre-training the teacher model well is necessary for CAFS to complete the segmentation task successfully.

### 5.3. Comparison with State-of-the-Art Models

We compare CAFS with four state-of-the-art models, including U-Net, U-Net with attention mechanism, Duo-SegNet, and TCSM. Among them, the U-Net and U-Net with attention mechanisms are the fully supervised methods. In addition, the Duo-SegNet [66] and TCSM [67] are the semi-supervised methods. In the validation process, we first train the model using the parameters recommended in the corresponding paper and subsequently analyze the performance of each method on the validation set. In the process, the same training set, test set, and validation set are used for all five methods and evaluate them by using the metrics in Section 4.3.

Table 5 shows the metrics performance of the five methods for nasopharyngeal carcinoma segmentation. The specific metrics values for each model are as follows: For U-Net, the DSC is 0.7456, which is 12.67% lower than CAFS, the Jaccard is 0.6868, which is 10.96% lower than CAFS, the precision is 0.6569, which is 22.8% lower than CAFS, the recall is 0.8822, which is 0.26% higher than CAFS. For U-Net with attentional mechanism, the DSC is 0.8198, which is 5.25% lower than CAFS, the Jaccard is 0.7011, which is 9.53% lower than CAFS, the precision is 0.8202, which is 6.46% lower than CAFS, the recall is 0.8309, which is 4.87% lower than CAFS. For Duo-SegNet, the DSC is 0.8130, which is 5.93% lower than CAFS, and the Jaccard is 0.6849, which is 11.15% lower than CAFS, the precision is 0.7966, which is 8.83% lower than CAFS, the recall is 0.8307, which is 4.89% lower than CAFS. For TCSM, the DSC is 0.7970, which is 7.53% lower than CAFS, and the Jaccard is 0.6987, which is 9.77% lower than CAFS, the precision is 0.8014, which is 8.35% lower than CAFS, the recall is 0.8978, which is 1.82% higher than CAFS. Among the metrics of these models, all metric values are highest for CAFS except for TCSM, whose recall is 5% higher than CAFS. These metrics data suggest that CAFS has higher segmentation efficacy for nasopharyngeal carcinoma than these models.

**Figure 4:**
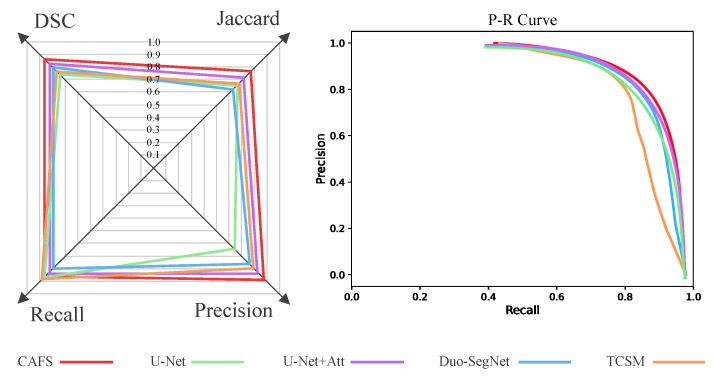
Radar plots and P-R curve. The radar plot is based on the four metrics of DSC, Jaccard, precision, and recall as axes. Each polygon corresponds to a model, and the four coordinate values of each model are the values of the four metrics. The closer the vertices of the polygon are to the outside, the better the model performs on the corresponding metrics. Each curve in the P-R curve corresponds to a model, and if the area enclosed under the curve is larger, it means that the corresponding model segmentation performs better.

To better compare the performance of each model, we plotted the contents of Table 3 as a radar chart (Figure 4) and drew a P-R curve. The radar plot takes DSC, Jaccard, Precision, and Recall as coordinates, and the more the coordinates are to the outside, the better the corresponding metric is. As can be seen, except for Recall, the other three coordinates of CAFS are all closer to the outside, indicating that CAFS works better. In the PR curve, if the curve of one model wraps the curve of the other model, the former outperforms the latter. Figure 4 shows that the red curve (CAFS) is in the uppermost right position, which indicates that CAFS performs better than the other four models. It is well known that U-Net is a fully supervised method specialized in the segmentation of medical images. In addition, CAFS uses fewer labeled data to achieve better results than the fully supervised methods. In addition, CAFS also achieves better performance than the semi-supervised methods, Duo-SegNet and TCSM.

To intuitively compare the segmentation effect of each model on nasopharyngeal carcinoma, we selected several representative nasopharyngeal carcinoma images in the test set and visualized the segmentation results in Figure 3. In general, the segmentation results of CAFS are closer to the ground truth. In addition, in the images of nasopharyngeal carcinoma that resemble the surrounding tissue (Figure 3 rows 1, 3, 7), the segmentation results of CAFS included the least amount of surrounding tissue, which indicates that CAFS is less likely to confuse nasopharyngeal carcinoma with the surrounding tissue. Moreover, in the images of nasopharyngeal carcinoma with complex boundaries (Figure 3 rows 2, 4, 5, 6), CAFS handles detailed segmentation of complex boundaries more accurately, which shows that CAFS is capable of handling segmentation tasks with complex boundaries.

## 6. Conclusions

This paper proposes a semi-supervised nasopharyngeal carcinoma segmentation method named CAFS. CAFS employs three strategies to overcome the difficulty of nasopharyngeal carcinoma segmentation. The teacher–student cooperative segmentation mechanism addresses the problem of difficulty to obtain labeled data for nasopharyngeal carcinoma, which allows CAFS to segment using only a small amount of labeled data. The attention mechanism addresses the problem of similarity of the nasopharyngeal carcinoma with surrounding tissues, which prevents the model from confusing them. The feedback mechanism allows CAFS to segment the boundaries of nasopharyngeal carcinoma more accurately. These three approaches have allowed CAFS to perform well in nasopharyngeal carcinoma segmentation.

We analyzed the segmentation performance of CAFS and explored the effect of the amount of labeled data in the training set on the final segmentation results. In addition, we did ablation analysis and proved that each part of our method is effective. Finally, we compare CAFS with state-of-the-art segmentation methods for nasopharyngeal carcinoma, and the results show that CAFS outperforms other methods.

At the same time, there are some limitations of CAFS. First, CAFS uses the framework of U-Net2D, while many medical data are now stored in volumetric, i.e., 3D, format. This means that, when segmenting 3D nasopharyngeal carcinoma data, the volumetric images need to be artificially converted into 2D images, which increases the time cost of segmentation. Second, the teacher model and the student model in CAFS have the same structure, which means that CAFS has to take up more computational resources than the model with only one network. Thus, our future work will focus on adapting CAFS to segmented volume medical data on the one hand, and simplifying the network structure while maintaining excellent performance on the other hand.

## Figures and Tables

**Table 1:** The main setting of hyperparameters for CAFS.

Optimizer	Weight Decay	Learning Rate	Training Iterations	Batch Size
SGD	0.0001	0.001	1500	8

**Table 2:** Segmentation performance of CAFS for nasopharyngeal carcinoma when different amounts of labeled data are provided for model training. The four metrics of DSC, Jaccard, precision, and recall were used to evaluate the performance of the segmentation. The highest value of each metric is bolded in the table. The data show that the best training results are achieved when 1000 labeled samples are provided for the training model.

Number of Labeled Data	DSC	Jaccard	Precision	Recall
500	0.8345	0.7234	0.8378	0.8469
**1000**	**0.8723**	**0.7964**	**0.8849**	**0.8796**
1500	0.8387	0.7207	0.8342	0.8479
2000	0.8420	0.7297	0.8413	0.8485

**Table 3:** Segmentation performance of the model fusing different attention mechanisms. Except for the replacement of the attention mechanism, the rest of the model and the parameter settings are identical to CAFS. The adaptive attention mechanism achieves the highest values on all three metrics: DSC, Jaccard, and Precision, indicating its superior feature extraction performance.

Attention Mechanism	DSC	Jaccard	Precision	Recall
**Ours**	**0.8723**	**0.7964**	**0.8849**	0.8796
CBAM [62]	0.8078	0.6303	0.6970	0.8682
SEAttention [63]	0.7986	0.6647	0.7258	**0.8876**
ECAAttention [64]	0.7924	0.6562	0.7187	0.8830
CoTAttention [65]	0.8200	0.6949	0.8639	0.7366

**Table 4:** Results of ablation experiments. Each abbreviation in the first column stands for C-S (Cooperative segmentation), Att (Attention mechanism), and Fb (Feedback mechanism). We use four metrics, DSC, Jaccard, precision, and recall, to evaluate the performance of segmentation with different combinations of modules.

Model	DSC	Jaccard	Precision	Recall
**C-S + Fb + Att**	**0.8723**	**0.7964**	**0.8849**	**0.8796**
C-S + Fb	0.8394	0.7632	0.8425	0.8487
C-S + Att	0.8579	0.7758	0.8562	0.8478
C-S	0.8385	0.7467	0.8312	0.8335
Without Pre-training	0.5935	0.4376	0.4230	0.5226

**Table 5:** Performance of CAFS and four other state-of-the-art models on the task of segmenting nasopharyngeal carcinoma. The training, testing, and validation processes for all five methods use the same data set distribution. We used four metrics to evaluate the performance of each model, DSC, Jaccard, precision, and recall, respectively. The higher the value of any metric, the better the model performs on the corresponding performance evaluation. The most optimal value of each metric is bolded in the table. It can be seen that, except for recall, the other three metrics of CAFS achieved the highest values.

Model	DSC	Jaccard	Precision	Recall
**CAFS**	**0.8723**	**0.7964**	**0.8849**	0.8796
U-Net [39]	0.7456	0.6868	0.6569	0.8822
U-Net + Att	0.8198	0.7011	0.8202	0.8309
Duo-SegNet [66]	0.8130	0.6849	0.7966	0.8307
TCSM [67]	0.7970	0.6987	0.8014	**0.8978**

## Data Availability

The data presented in this study are available on request from the corresponding author. The data are not publicly available due to patient privacy.
